# Pathway-based analysis of rare and common variants to test for association with blood pressure

**DOI:** 10.1186/1753-6561-8-S1-S101

**Published:** 2014-06-17

**Authors:** Huda Alsulami, Xiaofeng Liu, Joseph Beyene

**Affiliations:** 1 Statistics Department, King Abdulaziz University, Abdullah Sulayman, Jeddah 22254, Saudi Arabia; 2 Mathematics and Statistics Department, McMaster University, Hamilton, Ontario, Canada; 3 Population Genomics Program, Department of Clinical Epidemiology & Biostatistics, McMaster University, Hamilton, Ontario, Canada

## Abstract

Our goal is to test the effect of both rare and common variants in a blood pressure study. We use a pathway-based approach, gene-set enrichment analysis, to search for related genes affecting 4 phenotypes: systolic blood pressure, diastolic blood pressure, the difference between each of them and mean arterial pressure, which is a weighted linear combination of systolic and diastolic blood pressure. Using the real Genetic Analysis Workshop 18 data, we consider both rare and common variants in our analysis and incorporate other covariates by using a recently proposed test statistic.

Our study identified a commonly enriched gene set/pathway for the two derived phenotypes we analyzed: the difference between systolic and diastolic blood pressure and mean arterial pressure, but none is identified with the individual blood pressure phenotypes. The gene *CD47*, in the enriched gene pathway/set, was reported in previous studies to be related to blood pressure.

The findings are not surprising because the sample size we use in our analysis is small, and hence power to detect small but important effects is likely inadequate.

## Background

Worldwide, hypertension contributes to more than 10 million deaths and it affects one-third of the adult population per year [1]. It was predicted that the incidence of hypertension among adults in 2025 will reach 1.56 billion and contribute to approximately 54% of stroke and 47% of ischemic heart disease. Furthermore, it is a major risk factor for cardiovascular disease [2]. Several factors, including genetic, environmental, and demographic factors, play a major role in the development of hypertension. However, it is believed that 30% to 60% of the variability in blood pressure (BP) is inherited [1].

Many genome-wide association studies (GWAS) have been conducted to identify single-nucleotide polymorphism (SNPs) that are significantly associated with systolic blood pressure (SBP), diastolic blood pressure (DBP), and/or hypertension.

Meta-analysis findings of the Global BPgen (Global Blood Pressure Genetics) consortium (n = 34,433) and CHARGE (The Cohorts for Heart and Aging Research in Genome Epidemiology) consortium (n = 29,136) based on populations of European ancestry identified 4 loci significantly associated with SBP (ATP2B1, CYP17A1, PLEKHA7, SH2B3), 6 associated with DBP (ATP2B1, CACNB2, CSK-ULK3, SH2B3, TBX3-TBX5, ULK4), and 1 associated with hypertension (ATP2B1) [1]. However, a genome-wide association study by Adeyemo et al [3] based on a population of African Americans (n = 1017) identified significant loci for SBP in or near the genes *PMS1, SLC24A4, YWHA7, IPO7*, and *CACANA1H*, while no significant loci were discovered to be associated with DBP or hypertension.

Unlike single-gene analysis, pathway-based approaches consider multiple genes that are related together within gene sets/pathways; these pathways are predefined gene sets from biological databases. The aim of pathway-based approaches is to assess the significance of these sets/pathways by evaluating the enrichment of genes within a pathway at the top of a list of ranked genes [4-6]. Pathway-based analysis was originally applied to gene expression data; however, it has also been applied to GWAS data [4]. In this paper, we use a pathway-based approach based on Gene Set Enrichment Analysis (GSEA) [4]. We consider both rare and common variants and incorporate other covariates, including age, gender, use of antihypertensive medications, and smoking status. Our main focus is to test the effects of both rare and common variants on SBP, DBP, the difference between them (SBP-DBP), and mean arterial pressure (defined as MAP = [⅔ DBP] + [⅓ SBP]) by applying GSEA.

## Methods

### Phenotype and covariate data description

This data set was provided by the organizers of the Genetic Analysis Workshop 18 (GAW18). From this data set, we considered the 157 unrelated individuals with their phenotypes. Phenotypes were taken at 4 time points and included systolic and DBP measurements and hypertension. The following covariates were also provided: age, smoking status, antihypertensive medications usage, and gender. In our analysis, we used the baseline data; among the 157 unrelated individuals we had 129 individuals who had been genotyped. Table 1 summarizes the data.

**Table 1:** Descriptive statistics for phenotypes and covariates at baseline for 129 unrelated individuals

Variable	Summary measure*
SBP	128.4 ± 21.8
DBP	71.8 ± 9.2
MAP	90.7 ± 11.6
SBP-DBP	56.6 ± 19
Hypertension (Yes, No)	129 (29.5)
Age	52.9 ± 15.6
Sex (Female)	129 (60.5)
Medications use (Yes, No)	129 (20.2)
Smoking status (Yes, No)	129 (24.8)

*Mean ± SD for continuous variables; n(%) for categorical variables.

### Genotype data description

Genotype data were provided only for odd-numbered autosomal chromosomes. In this paper, we focus on variants on chromosome 3 (as suggested by the GAW18 organizers to allow comparisons of findings with other GAW18 contributions).

### Pathway-based analysis

We considered 4 phenotypes of interest SBP, DBP, SBP-DBP, and MAP and performed pathway-based analysis. We followed the following steps [4,5]:

#### Step 1: Mapping SNPs to genes

Among the 1,215,296 SNPs on chromosome three, 523,147 SNPs were mapped to 1224 known genes using NBCI2R.

#### Step 2: Obtaining test statistics for genes

We considered both rare and common variants and other covariates (age, smoking status, medications use, and gender) to assign a test statistic for each gene. VW-TOW (variable weight test for testing the effect of an optimally weighted combination of variants) [8] was used to construct test statistics and their *p *values. Assume that we have *n *individuals who have been genotyped at *M *variants and is the trait of interest for the *i*^th ^individual. Each individual has a genotypic score ^T ^where {0,1,2} denotes the number of copies of the minor allele for the *m*^th ^variant of the *i*^th ^individual. We used a minor allele frequency (MAF) threshold of less than 1% to define rare variants. To test the effect of the optimally weighted combination (TOW) of variants $x_{i}^{0}=\sum_{m=1}^{M}w_{m}^{0}x_{im}$, we used the statistic:

$$T_{T}=\overset{n}{\underset{i=1}{\sum }}\left(y_{i}-\overset{-}{y}\right)\left(x_{i}^{0}-{\bar{x}}^{0}\right)$$

where $w_{m}^{0}=\sum_{i=1}^{n}\left(y_{i}-\overset{-}{y}\right)\left(x_{im}-{\bar{x}}_{m}\right)/\sum_{i=1}^{n}{\left(x_{im}-{\bar{x}}_{m}\right)}^{2}$ are the optimal weights.

To test the effect of both rare and common variants, we applied TOW to each of them separately; $T_{r}$ and $T_{c}$ denote these statistics, respectively. Then we used the test statistic of VW-TOW:

$$T_{VW\text{\_}T}=\underset{0\leq \lambda \leq 1}{\text{min}}p_{\lambda }$$

where $p_{\lambda }$ is the *p *value of the test $T_{\lambda }$ and $T_{\lambda }=\lambda \frac{T_{r}}{\sqrt{var\left(T_{r}\right)}}+\left(1-\lambda \right)\frac{T_{c}}{\sqrt{var\left(T_{c}\right)}}$. To evaluate the *p *value of $T_{VW\text{\_}T}$, we used the permutation test.

We incorporated the other covariates $\left(z_{i1},\ldots ,z_{ip}\right)$^T ^for each individual *i *, by adjusting $y_{i}$ and $x_{im}$ using linear regression:

$$y_{i}=\alpha_{0}+\alpha_{1}z_{i1}+\ldots +\alpha_{p}z_{ip}+\varepsilon_{i}\text{ and}$$

$$x_{im}=\alpha_{0m}+\alpha_{1m}z_{i1}+\ldots +\alpha_{pm}z_{ip}+\tau_{im}$$

And by using the residuals ${\tilde{y}}_{i}$ and ${\tilde{x}}_{im}$, the following TOW and VW-TOW were used:

$$T_{TOW}=T_{T|y_{i}={\tilde{y}}_{i},x_{im}={\tilde{x}}_{im}}\text{ and}$$

$$T_{VW\text{\_}TOW}=T_{VW-T|y_{i}={\tilde{y}}_{i},x_{im}={\tilde{x}}_{im}}$$

#### Step 3: Pathway analysis

We ranked all the genes (N), that had *p *values and test statistics, based on their statistical significance from the smallest to the largest *p *values. From step 2, we had (N = 1187) genes for SBP and DBP and (N = 1188) genes for SBP-DBP and MAP. Using the GSEA method [6], we evaluated the significance of predefined gene sets/pathways obtained from online pathway databases (The Molecular Signatures Database) [9]. We used the c2 curated gene sets (v3.1), which are compiled from online pathway databases, publications in PubMed, and knowledge of domain experts [9], which consisted of 4850 gene sets, but we only considered 3638 sets that had at least 1 gene from chromosome 3 and at least 10 genes in total. Of the 3638 pathways, 69.1% have between 1 and 5 genes on chromosome 3, while 24.1% of the pathways have between 6 and 20 genes and 6.8% have between 21 and 140 genes on the same chromosome. Then we calculated the enrichment score (ES) for each set/pathway using a weighted Kolmogorov-Smirnov-like running-sum statistic. This statistic describes the overrepresentation of the genes within the set at the top of the ranked genes. We then adjusted for different sizes of genes using 1000 gene-based permutations (π) and calculated the normalized enrichment score (NES) for each set (*S*).

$$\text{NES}\left(S\right)=\frac{Actual\left(ES\left(S\right)\right)}{mean\left(ES\left(S,\pi \right)\right)}$$

To estimate the significance level of NES for each set/pathway, we used the gene-based permutation approach to obtain the empirical *p *values of the NES. We used 1000 gene-set permutations and then we considered the set/pathway to be significantly enriched if its false discovery rate (FDR) *q *value is less than 0.05. We implemented the analysis using the GSEApreranked tool included in the GSEA software [6,7].

## Results

Considering common and rare variants from chromosome 3 with other covariates, and applying GSEA to our data, we ranked the top 10 gene sets/pathways based on their FDR *q *values for each phenotype. These ranked genes are listed in Tables 2, 3, 4, and 5 for MAP, (SBP-DBP), SBP, and DBP phenotypes, respectively. We found that no gene sets were enriched when we considered SBP or DBP. However, we were able to identify 1 significant enriched gene pathway from c2 curated gene sets (Table 2) with MAP. Interestingly, the same pathway was declared to be significantly enriched with the difference between SBP and DBP phenotype (Table 3). We identified the same gene pathway (Koyama_Sema3B_Targets_DN) in both phenotypes, and this pathway had been shown to be related to different kinds of cancer [10,11]. In this pathway, 12 of 18 genes on chromosome 3 contributed to the enrichment score and the most interesting gene in this pathway is CD47. Several articles [12,13] reported that this gene regulates BP.

**Table 2:** The top 10 gene sets/pathways from c2 curated gene sets ranked by FDR *q *values for MAP

Pathway name	No. genes*	ES	NES	FDR *q *value
KOYAMA_SEMA3B_TARGETS_DN	18	0.611	2.226	0.040

HUANG_GATA2_TARGETS_UP	11	0.559	1.769	0.903

ONO_FOXP3_TARGETS_DN	5	0.724	1.757	0.929

BENPORATH_ES_2	4	0.859	1.938	0.967

CARDOSO_RESPONSE_TO_GAMMA_RADIATION_AND_3AB	3	0.865	1.770	0.980

ZHANG_TLX_TARGETS_60HR_UP	22	0.423	1.625	0.981

LAIHO_COLORECTAL_CANCER_SERRATED_DN	1	0.891	1.202	0.992

PID_INTEGRIN2_PATHWAY	1	0.915	1.203	0.992

LIU_TARGETS_OF_VMYB_VS_CMYB_DN	5	0.501	1.203	0.992

LIM_MAMMARY_STEM_CELL_DN	21	0.317	1.203	0.992

*Number of genes on chromosome 3.

**Table 3:** The top 10 gene sets/pathways from c2 curated gene sets ranked by FDR q-values for the difference between SBP and DBP

Pathway name	No. genes*	ES	NES	FDR *q *value
KOYAMA_SEMA3B_TARGETS_DN	18	0.614	2.227	0.042
ZHAN_MULTIPLE_MYELOMA_CD1_AND_CD2_UP	4	0.830	1.867	0.822
TURASHVILI_BREAST_LOBULAR_CARCINOMA_VS_LOBULAR_NORMAL_UP	7	0.691	1.888	0.823
BREDEMEYER_RAG_SIGNALING_NOT_VIA_ATM_DN	4	0.811	1.844	0.871
LI_INDUCED_T_TO_NATURAL_KILLER_DN	7	0.693	1.907	0.892
BENPORATH_ES_2	4	0.858	1.941	0.917
TONKS_TARGETS_OF_RUNX1_RUNX1T1_FUSION_SUSTAINED_IN_GRANULOCYTE_UP	3	0.869	1.762	0.958
CHANG_CORE_SERUM_RESPONSE_DN	21	0.290	1.091	0.976
WONG_ENDMETRIUM_CANCER_UP	2	0.625	1.082	0.976
NOUZOVA_TRETINOIN_AND_H4_ACETYLATION	18	0.299	1.090	0.976

*Number of genes on chromosome 3.

**Table 4:** The top 10 gene sets/pathways from c2 curated gene sets ranked by FDR *q *values for SBP

Pathway name	No. genes*	ES	NES	FDR *q *value
GOTTWEIN_TARGETS_OF_KSHV_MIR_K12_11	6	0.588	1.554	0.623
SMIRNOV_RESPONSE_TO_IR_6HR_UP	6	0.583	1.554	0.628
PID_SHP2_PATHWAY	3	0.738	1.542	0.629
JIANG_VHL_TARGETS	6	0.588	1.550	0.630
SHEDDEN_LUNG_CANCER_GOOD_SURVIVAL_A12	18	0.408	1.555	0.631
PLASARI_TGFB1_SIGNALING_VIA_NFIC_10HR_UP	5	0.628	1.543	0.632
CHEN_PDGF_TARGETS	4	0.668	1.545	0.632
PID_IGF1_PATHWAY	3	0.759	1.542	0.633
NAKAMURA_TUMOR_ZONE_PERIPHERAL_VS_CENTRAL_DN	32	0.349	1.555	0.633
PID_BCR_5PATHWAY	3	0.764	1.557	0.633

*Number of genes on chromosome 3.

**Table 5:** The top 10 gene sets/pathways from c2 curated gene sets ranked by FDR *q *values for DBP

Pathway name	No. genes*	ES	NES	FDR *q *value
PHONG_TNF_RESPONSE_VIA_P38_COMPLETE	13	0.583	1.743	0.580
DELYS_THYROID_CANCER_DN	11	0.612	1.748	0.599
CORRE_MULTIPLE_MYELOMA_DN	3	0.897	1.733	0.603
SHEPARD_BMYB_MORPHOLINO_UP	10	0.609	1.712	0.617
LI_INDUCED_T_TO_NATURAL_KILLER_UP	17	0.537	1.724	0.617
WILCOX_PRESPONSE_TO_ROGESTERONE_UP	6	0.705	1.700	0.621
WAMUNYOKOLI_OVARIAN_CANCER_LMP_DN	13	0.562	1.674	0.632
KEGG_RENIN_ANGIOTENSIN_SYSTEM	3	0.932	1.761	0.632
OSWALD_HEMATOPOIETIC_STEM_CELL_IN_COLLAGEN_GEL_DN	11	0.605	1.751	0.633
REACTOME_POST_TRANSLATIONAL_PROTEIN_MODIFICATION	16	0.535	1.685	0.644

*Number of genes on chromosome 3

Because our pathway-based analysis is restricted to genes on chromosome 3, the number of pathways used for analysis exceeded the number of genes, which can have important implications in interpreting our findings. The results from our analyses should be interpreted cautiously.

## Conclusions

Gene-set enrichment analysis considers multiple genes that are related biologically. In our data, we identified 1 identical enriched gene set/pathway with the MAP and the difference between SBP and DBP. The gene CD47 in this pathway was reported previously to be related to BP.

Our analysis included only 129 unrelated individuals. Sample size plays a major role in identifying enriched gene sets/pathways, which could explain the lack of significant pathways in our analysis. Future studies can be done by applying GSEA on large family-based data where incorporating both rare and common variants, taking into account the correlations between individuals and increasing the sample size, may lead to new discoveries.

## Competing interests

The authors declare that they have no competing interests.

## Authors' contributions

HA designed the overall study; HA and XFL performed all of the data analysis. HA and JB drafted the manuscript and JB conceived the study and provided critical comments. All authors read and approved the final manuscript.
